# The Dark Corner of the Pituitary Gland: A Case Report and Literature Review of Primary Melanocytoma

**DOI:** 10.2174/0115734056359260250623115500

**Published:** 2025-06-30

**Authors:** Jiajing Ni, Jianhua Wang

**Affiliations:** 1 Health Science Center, Ningbo University, Ningbo 315211, China; 2 Department of Radiology, The First Affiliated Hospital of Ningbo University, Ningbo 315010, China; 3 Department of Radiology, The First Affiliated Hospital of Xiamen University, Xiamen 361000, China

**Keywords:** Primary pituitary melanocytoma, Pituitary tumor, Melanocytoma, Pituitary melanocytoma imaging characteristics, Melanin, CT, CNS

## Abstract

**Background:**

Primary pituitary melanocytoma, an exceedingly rare tumor, may resemble pituitary adenoma with apoplexy owing to its heterogeneous melanin concentration and possible hemorrhagic events. An accurate diagnosis of melanocytoma is, therefore, essential.

**Case Presentation:**

We present a case of a 31-year-old female patient who exhibited a progressively worsening headache that commenced one month prior. MRI showed a significantly enlarged sella turcica with a gourd-shaped lesion that had a mixture of short T1 and T2 signals. In conjunction with the MRI findings, CT scans, both non-contrast and contrast-enhanced, revealed a circular, dense region in the sellar area, exhibiting heightened enhancement post-contrast administration. Subsequently, this patient was scheduled for endoscopic transnasal skull base tumor resection and skull base reconstruction. Later, histopathological assessment showed red-S-100 (+), red-melanin A (+), red-KI-67 (+5%), red-melanoma (+), P53 (+), red-P53 (+) and Ki-67 (+) and suggested an intermediate-grade melanocytoma, positioning this lesion between benign and malignant on the spectrum of melanocytic neoplasms.

**Conclusion:**

This case report evaluated the presentation, key imaging findings, and histopathological features that help differentiate primary melanocytoma from other tumors and discussed key management and prognostic considerations following diagnosis.

## 1 INTRODUCTION

Primary melanocytomas of the Central Nervous System (CNS) represent an exceedingly rare subset of neoplasms, with an estimated annual incidence of merely 0.01 per 100,000 individuals. These elusive tumors account for 1% of all melanomas and a mere 0.07% of all brain tumors [1]. Among this already rare category, primary pituitary melanocytomas-those originating solely from the pituitary gland-present a notably significant diagnostic hurdle. In particular, it is difficult to differentiate a primary sellar melanocytic tumor from a pituitary metastasis of melanoma clinically, radiologically, and histologically. According to the Hayward proposition, the following factors are significant: no malignant melanoma outside of the CNS, involvement of the sellar leptomeninges, and a single sellar lesion. Thus, a primary sellar melanocytoma is established only after the exclusion of secondary metastatic disease from a cutaneous, mucosal, or retinal primary tumor, in addition to histopathologic confirmation of the sellar lesion [2]. In addition, histology, Magnetic Resonance Imaging (MRI), and Computed Tomography (CT) have an important role in the diagnosis of melanoma [2, 3]. However, at present, no evidence-based guidelines exist for the diagnosis of primary pituitary melanocytoma.

This case report documents an exceptional instance of a primary pituitary melanocytoma in a patient without any antecedent history of cutaneous melanoma. This uncommon case aims to enhance the sparse literature on these tumors, highlighting their rarity and the complex diagnostic considerations they need. Furthermore, this report serves to heighten awareness among clinicians and pathologists about the possibility of encountering such lesions, even in the absence of a primary melanoma elsewhere in the body.

## 2 CASE PRESENTATION

The patient provided informed consent for this case report. All procedures were approved by the Medical Ethics Committee, the First Affiliated Hospital of Ningbo University, and were conducted according to the 1975 Declaration of Helsinki (revised in 2013) (Registration number: MR-33-25-002961). A 31-year-old female patient presented with a steadily increasing headache that commenced one month prior without accompanying symptoms or identifiable causative circumstances. Her medical history was unremarkable, with no pre-existing health conditions, no significant long-term sun exposure, no prior surgeries involving the skin, mucous membranes, or eyes, and no family history of cancer. Upon physical examination, the patient appeared to be in good overall health. Her clinical status was assessed using several standardized measures. Her Activities of Daily Living (ADL) score was one hundred at admission, indicating full independence in daily activities. The Caprini score for Venous Thromboembolism (VTE) risk was very low, suggesting a minimal risk for VTE. Psychological evaluation using the Brief Psychosomatic Health Questionnaire (BPSRQ) yielded a score of 0, indicating normal psychological health. A comprehensive panel of hematological tests was conducted, including complete blood count, electrolyte levels, erythrocyte sedimentation rate, and liver enzyme profile.

Additionally, tumor markers, such as serum alpha-fetoprotein, human chorionic gonadotropin, placental alkaline phosphatase, and lactate dehydrogenase were measured. All laboratory results were within normal reference ranges, providing no immediate indication of underlying systemic disease or malignancy. This comprehensive initial assessment established the foundation for additional diagnostic inquiries, considering the persistent nature of the patient's headache despite an otherwise normal clinical presentation.

### 2.1 Diagnostic Imaging

**Fig. (2):**
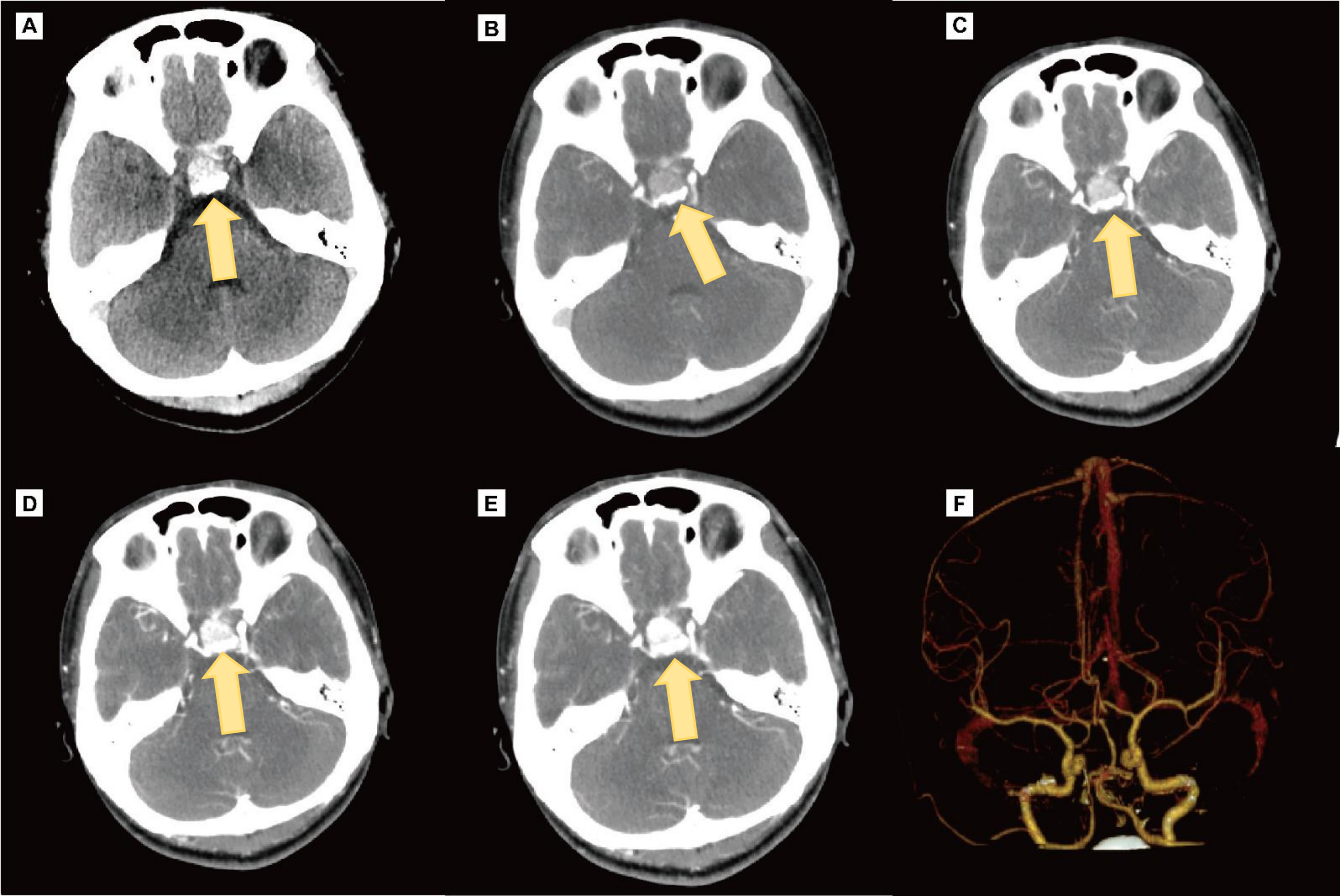
Preoperative Intracranial Artery CTA. (**A**) Coronal CT plain scan shows a round high-density area in the sellar region (as indicated by the arrow). (**B**, **C**, **D**, and **E**) Coronal CT-enhanced scans reveal enhancement within the lesion (as indicated by the arrow). (**F**) 3D reconstruction of computed tomography angiography (CTA) shows no abnormalities.

Upon admission, the patient underwent a comprehensive series of diagnostic imaging studies to evaluate the underlying cause of her persistent headache. These included non-contrast and contrast-enhanced head CT scans, non-contrast and contrast-enhanced head and pituitary MRI, as well as CT angiography (CTA) and CT venography (CTV). The MRI findings were particularly significant, revealing a markedly enlarged sella turcica containing a gourd-shaped lesion. This lesion exhibited mixed short T1 and short T2 signals and measured approximately 16x15 mm. Additionally, it showed hyperintensity on T1-weighted images and hypointensity on T2-weighted images. But its lesion sites were uncommon. The normal pituitary structure was obscured, and there was evident compression and elevation of the optic chiasm, although the cavernous sinuses appeared uninvolved. Upon contrast administration, the lesion demonstrated slight punctate enhancement Fig. (**1A**-**D**). Complementary to the MRI findings, both non-contrast and contrast-enhanced CT scans depicted a round, high-density area within the sellar region, which showed enhancement following contrast administration Fig. (**2A**-**E**). Notably, the CTA and CTV scans did not reveal any vascular abnormalities (Fig **2F**).

### 2.2 Histopathological Analysis

The patient was scheduled for endoscopic transnasal skull base tumor resection and skull base reconstruction. After the operation, immunohistochemical analysis was performed at the Ningbo Clinical Pathology Diagnosis Center using histological sections previously fixed in 10% buffered formalin and embedded in paraffin. The BenchMark ULTRA platform was employed using monoclonal or polyclonal antibodies in combination with Ventana's detection system, following these steps: deparaffinization, antigen retrieval, blocking of non-specific reactions, incubation with the described panel of antibodies, detection, and amplification (if necessary), and development. Individualized positive controls were used to demonstrate the fidelity of the reactions. Immunohistochemistry results showed Red-S-100 (+), Red-Melan A (+), Red-KI-67 (+5%), Red-Melanoma (+), P53 (+), Red-P53 (+), and Ki-67 (+).This immunohistochemical profile, particularly the positivity for melanocytic markers (S-100, Melan A, and Melanoma) in conjunction with a low Ki-67 proliferation index of 5%, led to the diagnosis of a pituitary melanocytoma. The level of Ki-67 directly reflects the active level of tumor cell proliferation. The higher the Ki-67 value, the more active the proliferation of the tumor cells and the higher the degree of malignancy and progression of the tumor. The low Ki-67 index of this patient indicated a less aggressive nature on the spectrum of melanocytic neoplasms Fig. (**3A**-**E**).

### 2.3 Outcome and Follow-up

The patient's post-operative recuperation proceeded without complications, resulting in the full alleviation of the initial headache. Given the unusual nature of the diagnosis, a comprehensive evaluation was undertaken to rule out other potential primary sites of melanoma. A dermatologist performed a comprehensive assessment, discovering no melanotic lesions present on the skin, ocular regions, or other mucosal surfaces. Further investigations included imaging studies of the chest and abdomen, esophagogastroduodenoscopy, and colonoscopy, all of which revealed no abnormalities. An ophthalmologic examination was also performed, conclusively ruling out any form of uveal melanoma. Based on these extensive findings, coupled with the histopathological results, the final diagnosis was confirmed as a primary pituitary melanocytoma. At the six-month postoperative follow-up, the patient showed no clinical signs of significant recurrence. However, MRI monitoring indicated a slight possibility of residual tumor tissue. As a result, the patient was placed on a regimen of regular imaging examinations to closely monitor for any signs of tumor recurrence or progression. Taken together, symptoms and signs of the patient and MRI play a significant role in the disease monitoring process of primary melanoma.

## 3 DISCUSSION

In our comprehensive research on pituitary melanocytoma, we systematically searched academic databases, such as PubMed and Web of Science, using keywords like “pituitary melanocytoma,” “melanoma,” and “pituitary tumor.” This research yielded fourteen studies related to primary pituitary melanocytoma and primary sellar melanocytoma. These studies included clinical information, imaging findings, and treatment details for each reported case (Tables **1** and **2**). The inclusion criteria required that cases be confirmed as originating from the pituitary gland through imaging and pathological analysis, with comprehensive clinical, diagnostic, and treatment data. Cases involving metastatic melanomas or melanomas of unknown primary origin were excluded.

### 3.1 Origin

The developmental origin of melanocytes can be traced back to the neural crest during the early embryonic stages. From this primordial structure, melanocyte precursors migrate to various destinations, including the mucosal surfaces of the skin, visceral organs, uvea, and leptomeninges [4]. Within the Central Nervous System (CNS), melanocytomas are predominantly found in the posterior fossa and upper spinal cord, regions characterized by a high concentration of leptomeningeal melanocytes. Pituitary melanocytomas, nonetheless, constitute a fascinating deviation from this established distribution pattern. From an embryological perspective, two principal hypotheses have been proposed to explain the origin of primary melanocytomas in the sellar region:

#### 3.1.1 Meningeal Melanocyte Focus Malignancy Hypothesis

This theory posits that these tumors may arise from the malignant transformation of primary meningeal melanocyte foci. This hypothesis is rooted in the well-established embryonic origin of melanocytes from the neural crest [5].

#### 3.1.2 Intrinsic Brain and Pituitary Melanocytes Hypothesis

Fetisov *et al.* [6] reported the presence of functionally active melanocytes in the brain and pituitary of rats. While this finding has not been conclusively demonstrated in humans, it opens up the possibility of melanocytomas originating from resident melanocytes within these structures. The proposed hypotheses offer a structured approach to comprehending the rare manifestation of melanocytomas within the pituitary gland, an area not conventionally linked to elevated melanocyte densities. Further research is needed to elucidate the precise mechanisms underlying the development of these rare neoplasms in the sellar region.

### 3.2 Diagnosis

#### 3.2.1 Clinical Presentation

Primary pituitary melanocytoma is extremely rare, with only fourteen cases reported in the literature to date, making our case the 15th reported instance (Tables **1** and **2**). Most patients present with neurological symptoms or endocrine disorders, with only one case incidentally discovered during radiological examination [7]. Progressive vision impairment was reported in ten out of fifteen cases (66.67%), and various degrees of pituitary hormone secretion abnormalities were observed in thirteen out of fifteen cases (86.67%). Some patients exhibited mildly elevated serum prolactin levels due to compression of the pituitary stalk, leading to galactorrhea and amenorrhea. Additional symptoms included headaches and weakness.

#### 3.2.2 Imaging Findings

Primary pituitary melanocytomas generally manifest as round, nodular lesions on CT scans, characterized by high-density or iso-density. These lesions may include cystic or necrotic regions, though calcification is an uncommon finding [8]. MRI signals for these tumors are variable. Isiklar *et al*. [9]classified primary pituitary melanocytomas into four types:

##### 3.2.2.1 Melanin Type

It is categorized by high signal on T1-weighted images (T1WI), low signal on T2-weighted images (T2WI), and iso- or high signal on proton density images.

##### 3.2.2.2 Non-Melanin Type

It is categorized by low or iso signal on T1WI, high or iso signal on T2WI, and proton density images.

##### 3.2.2.3 Uncertain or Mixed Type

It is categorized by mixed signals different from the previous two types.

##### 3.2.2.4 Hemorrhagic Type

It shows hemorrhagic signals in various phases. The first and fourth types are the most common, accounting for about 70% of melanocytomas. Owing to the significant vascularity of melanocytomas and their origin in the neural crest, which possesses the capacity to differentiate into components of the vascular system, these tumors have the potential to disseminate through the subarachnoid space and perivascular spaces, resulting in vascular pigmentation. MRI with contrast enhancement typically shows marked tumor enhancement, and some cases may show dural enhancement in contact with the tumor, potentially confusing it with meningioma. Primary pituitary melanocytomas do not have characteristic CT features, and only melanin-type melanocytomas show a specific high signal on T1WI among pituitary tumors on MRI. In addition, positron emission tomography with 2-deoxy-2-[fluorine-18] fluoro-D-glucose integrated with computed tomography (18F-FDG PET/CT) has emerged as a powerful imaging tool for the detection of various tumors, such as melanocytoma. Therefore, 18F-FDG PET/CT can be used as a tool for melanocytoma diagnosis in future studies.

###### 3.2.2.4.1 Similar Morphological Features

Pituitary melanocytomas and pituitary adenomas may present similar morphological features on MRI. When melanocytomas do not exhibit typical pigmentation, their imaging characteristics may be indistinguishable from those of pituitary adenomas [10] Table **3**.

###### 3.2.2.4.2 Higher Frequency of Pituitary Adenomas

Pituitary adenomas are the most common tumor type in the pituitary region, and their clinical frequency far exceeds that of melanocytomas.

Consequently, practitioners frequently prioritize pituitary adenomas initially, drawing upon both empirical knowledge and statistical likelihood. Among the fourteen cases we collected, four were initially diagnosed as pituitary adenomas. Additionally, primary pituitary melanocytomas are difficult to distinguish from metastatic pituitary melanocytomas on imaging and histology. Only by excluding metastatic tumors can a diagnosis of primary pituitary melanocytoma be made.

#### 3.2.3 Histological Features

A definitive diagnosis is established through histological examination, identifying cytoplasmic melanin, immunohistochemical profiles, and ultrastructural characteristics. Lach *et al*. [11] detailed an amelanotic melanocytoma that required electron microscopy to confirm its melanocytic origin. Contemporary diagnostic biomarkers play a crucial role in the identification and characterization of melanocytic tumors. These include Melan-A, HMB-45, tyrosinase, and MITF, which are typically specific to melanocytic neoplasms [12]. However, it is important to note that although these markers confirm melanocytic lineage, they do not inherently indicate metastatic potential [13]. In contrast, SOX10 has emerged as a reliable marker for detecting metastatic melanoma [14].

The assessment of proliferative activity is essential in distinguishing malignant from benign melanocytic lesions. Two key parameters are commonly used for this purpose: the mitotic count, evaluated per ten high-power fields (HPF), and the Ki-67 labeling index, a proliferation marker expressed throughout the cell cycle. Melanocytomas are typically characterized by a low mitotic index (≤ 1 per 10 HPF) and a low Ki-67 labeling index (≤ 2%). In contrast, melanomas usually exhibit a higher mitotic index (≥ 5 per 10 HPF) and a higher Ki-67 labeling index (> 2%).

These proliferative indices serve as critical criteria in the histological grading and prognostic assessment of melanocytic tumors [15] (Tables **4** and **5**). Furthermore, the past few years have yielded an abundance of insights concerning the genetic modifications present in diverse melanocytic tumors. A study indicated that metastasized uveal melanomas exhibited mutations in GNAQ and BAP1. Therefore, BAP1 and GNAQ are important markers for the differential diagnosis of melanocytic tumors.

### 3.3 Treatment

Currently, there are no established guidelines for treating primary pituitary melanocytoma. In clinical practice, patients are often initially misdiagnosed with pituitary adenomas and placed on hormone replacement therapy. However, due to disease progression, these patients typically undergo transsphenoidal surgical resection of the tumor. Complete tumor resection is often unachievable, as it risks damaging the diaphragma sellae or cavernous sinus [2].

As a result, current literature suggests that the optimal treatment strategy involves a multimodal approach combining Gross Total Resection (GTR) with adjuvant radiotherapy (RT) and systemic therapy [2]. Evidence indicates that radiotherapy may be effective for long-term control of primary central nervous system (CNS) melanocytomas. Gamma Knife radiosurgery may be an appropriate option for managing residual or recurrent lesions following partial resection, provided there is no evidence of meningeal spread.

The role of chemotherapy in primary CNS melanocytomas remains unclear. Temozolomide is the most commonly used drug due to its ability to penetrate the blood-brain barrier and is often administered as monotherapy. Immunotherapy agents, such as vemurafenib, are used for metastatic melanoma; however, their efficacy in crossing the blood-brain barrier is still uncertain. Therefore, managing primary pituitary melanocytomas requires a tailored approach that considers the patient’s clinical presentation, tumor characteristics, and the potential benefits and risks of available treatment modalities.

### 3.4 Prognosis

The prognosis of primary pituitary melanocytomas varies, with nearly half of patients surviving beyond two years of follow-up (Table **2**). Research suggests that the outlook for primary pituitary melanocytomas is generally better than that of metastatic pituitary melanomas. Nevertheless, as shown in previous cases, the patient’s overall health and the tumor’s aggressive characteristics play crucial roles in determining prognosis. Although repeated surgeries and radiotherapy are often necessary, their effectiveness may be limited due to rapid tumor growth and high invasiveness [16-18]. It is important to note that poor outcomes in some patients are primarily due to comorbid conditions rather than the tumor itself. For example, severe complications, such as mitral rheumatism [19] or brain edema with herniation [17], have been reported as major contributors to unfavorable prognoses in certain cases. Therefore, a thorough evaluation of the patient’s overall health is essential during treatment and management to improve outcomes and increase survival rates.

## 4 SUMMARY

The rarity of primary pituitary melanocytoma is a significant aspect of this case report, with diagnosis primarily achieved through histological examination. Patients often present with pituitary tumor syndrome and/or anterior pituitary insufficiency, which can lead to misdiagnosis as non-secreting hemorrhagic pituitary adenomas. Thorough diagnostic evaluations are essential to rule out metastatic melanoma involving the pituitary gland. Surgical resection remains the primary treatment modality despite the challenges posed by the tumor’s location. Complete resection is often unattainable due to the complex anatomy of the sellar region. Consequently, postoperative Stereotactic Radiosurgery (SRS) is frequently necessary to address residual tumors and reduce recurrence risk. A multimodal approach, potentially including adjuvant therapies, may be required based on individual case factors. The prognosis of primary pituitary melanocytoma is characterized by a high recurrence rate, including the potential for late recurrences years after initial treatment. This necessitates prolonged and diligent monitoring through routine imaging and endocrine assessments. Close surveillance is essential for the early detection of tumor recurrence or progression. The management of primary pituitary melanocytoma demands a structured, multidisciplinary approach.

## CONCLUSION

This research provides insight into primary pituitary melanocytoma, emphasizing its rare occurrence in the sellar region. This case highlights the necessity of a multidisciplinary approach, integrating clinical, radiological, and histopathological knowledge for accurate diagnosis and personalized treatment. Although it remains a challenging condition, advancements in diagnostic techniques, surgical methods, and adjuvant therapies offer hope for improved outcomes. Ongoing research and accumulating clinical experience are vital for refining treatment strategies and enhancing prognostic accuracy for patients affected by this rare tumor.

## Figures and Tables

**Fig. (1):**
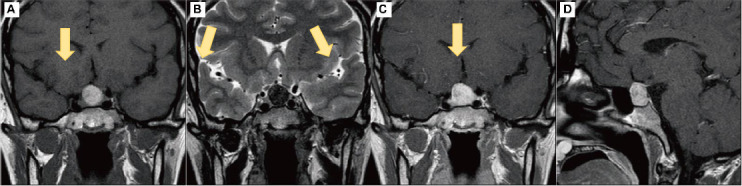
Preoperative Pituitary MRI Plain and Enhanced Scans. (**A**) Coronal MRI T1-weighted and (**B**) T2-weighted images show a pituitary lesion with an unclear pituitary stalk and an enlarged sella turcica (as indicated by the arrow). (**C**) Coronal and (**D**) sagittal T1-enhanced images display mild enhancement with small patchy non-enhancing areas within the lesion (as indicated by the arrow).

**Fig. (3):**
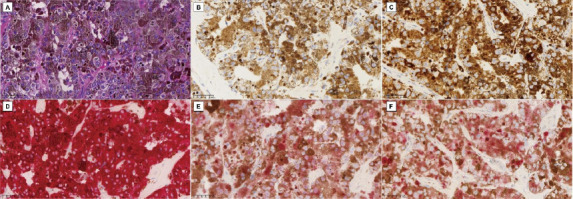
Postoperative pathology results. (**A**) HE staining shows irregular nuclear staining, sparse stroma, and densely packed cells (400x). (**B**) Cam5.2 immunohistochemistry shows positive expression of low-molecular-weight cytokeratin (400x). (**C**) Synaptophysin staining is positive (400x). (**D**) Melan-A staining is positive (400x). **(E**) p53 staining shows a pattern suggestive of mutation or overexpression (400x). (**F**) Ki-67 labeling indicates a low proliferation index (400x).

**Table 1:** Clinical information and imaging findings of all reported cases in the literature to date.

**Case**	**Year\Refs.**	**Age (years)**	**Gender**	**Clinical Symptoms**	**Imaging Modality**	**Tumor Location**	**Tumor Size (mm)**	**Invasion**	**Enhancement**
1	1963 [7]	62	M	None	MRI	Pituitary	Significantly enlarged	Erosion of sellar floor, compressing the left optic tract	-
2	2015 [10]	31	F	Amenorrhea for two years	MRI	Sella turcica	24x18x16 mm	Extending upward without cavernous sinus invasion	Mild homogeneous enhancement
3	2017 [11]	50	M	Transient aphasia, unrelated facial nerve palsy	MRI	Sella and suprasellar	~28 mm	Well-demarcated from adjacent tissues	Marked enhancement
4	2016 [16]	36	F	Headache, visual impairment	MRI	Sella and suprasellar	-	Peduncle displacement, infiltration of the optic tract	Heterogeneous enhancement
5	1997 [17]	47	M	Headache, endocrine dysfunction	CT, MRI	Sella turcica	~25 mm	Erosion of sellar floor, extending into sphenoid sinus and pre-chiasmatic cistern	-
6	2006 [18]	63	F	Headache, diplopia, bilateral ptosis	MRI	Pituitary	-	Extending into both cavernous sinuses	Non-enhanced area (4x5mm)
7	1976 [19]	54	M	Headache, visual impairment	CT	Sella turcica	-	Erosion of dorsum sella, extending left and anteriorly	-
8	2011 [20]	62	F	Visual impairment	MRI	Sella turcica	22x14x16 mm	Extending upward, compressing the optic chiasm	Marked enhancement
9	2013 [21]	82	F	Bilateral ophthalmoplegia	MRI	Sella turcica	-	Extensive invasion of both cavernous sinuses	Marked enhancement
10	1990 [22]	35	F	Oligomenorrhea, intermittent galactorrhea, headache	MRI	Sella turcica	-	Extending upward, compressing the left optic pathway	-
11	2004 [23]	37	F	Visual decline	MRI	Intrasellar and suprasellar	-	Pituitary and infundibulum involvement	Mild enhancement
12	2023 [24]	35	F	Oligomenorrhea, decreased libido	MRI	Suprasellar	22x14x16 mm	Compressing the optic chiasm	Marked enhancement
13	2023 [24]	51	M	Visual decline	MRI	Sella turcica	40x40x40 mm	Extending suprasellar and parasellar, compressing the optic chiasm	-
14	2009 [25]	61	F	Fatigue, progressive bitemporal hemianopia	MRI	Sella turcica	22x14x16 mm	Extending upward, compressing optic chiasm, no cavernous sinus invasion	Marked enhancement
15	2024	31	F	Headache	MRI	Pituitary	16x15 mm	Compressing and elevating optic chiasm, no cavernous sinus invasion	Mild enhancement

**Abbreviations:** M: Male ;F: Female ; CT: Computed Tomography ;MRI: Magnetic Resonance Imaging;

**Table 2:** Diagnostic and treatment information of all reported cases in the literature to date.

**Case**	**Year**	**Biological Findings**	**Initial Diagnosis and Treatment Plan**	**Surgical Situation and Disease Progression**	**Final Treatment Plan**	**Prognosis**
1	1963 [7]	Hypopituitarism	-	Two tumors in the pituitary, melanoma and chromophobe adenoma	Resection	Died after one year
2	2015 [10]	Gonadotropin, LH, FSH, ACTH deficiency, and hyperprolactinemia	-	Extensive bleeding in the initial surgery and highly vascular melanocytoma reoccurred after 6 years	Resection (40%)	Good
3	2017 [11]	Hypopituitarism	-	Negative melanocytoma markers, the melanotic variant in the sellar region	Subtotal resection, radiotherapy (25 Gy/5 sessions)	Good
4	2016 [16]	Mild hyperprolactinemia, hypothyroidism, and cortisol deficiency	-	Extensive tumor infiltration, adhesion, and congestion. Hypothalamic syndrome weeks later, carcinomatous meningitis after 6 months	Surgical resection, chemotherapy (BRAF V600 mutation-positive, vemurafenib, temozolomide second line), radiotherapy (30 Gy, over 4 weeks)	Died after 14 weeks
5	1997 [17]	Secondary adrenal insufficiency	-	Post-surgery radiotherapy, tumor recurrence, death from brain edema and herniation	Resection, radiotherapy (6 weeks, 6000 cGy)	Died two months post-surgery
6	2006 [18]	Normal	-	Blindness six weeks post-admission, MRI showed tumor enlargement and invasion of cavernous sinus.	Resection, radiotherapy	Died within months post-surgery
7	1976 [19]	-	-	The condition worsened post-surgery, died two weeks later, a tumor invasion of critical brain areas	Resection	Died post-surgery
8	2011 [20]	Hypopituitarism and hyperprolactinemia	Large pituitary adenoma; hormone replacement, dopamine agonists	Bilateral hemianopia	Resection, radiotherapy	Good
9	2013 [21]	Normal	-	Pink tumor, invasion of cavernous and sphenoid sinuses, partial resection via endoscopic assistance	Resection (50%), radiotherapy	NA
10	1990 [22]	Hyperprolactinemia	Prolactin-secreting adenoma	Tumor hemorrhage	Resection	NA
11	2004 [23]	Hypothyroidism, hypogonadism	-	MRI showed heterogeneous lesions, rapid vision deterioration	Resection, adjuvant radiotherapy (stereotactic fractionated, 1.8 Gy, total 40.4 Gy)	Good
12	2023 [24]	Slightly elevated prolactin (PRL) levels	Pituitary tumor; hormone replacement therapy	Condition progression (bilateral hemianopia)	Resection	Good
13	2023 [24]	Complete hypopituitarism	-	Tumor invasion of the left cavernous sinus, heavy bleeding, partial resection	Subtotal resection, radiotherapy (Gamma Knife radiosurgery, 15 Gy, 50% isodose)	Good
14	2009 [25]	Hypopituitarism, hyperprolactinemia	Non-secreting pituitary adenoma, hydrocortisone, and levothyroxine replacement therapy, dopamine agonists	Condition progression (bilateral hemianopia), fibrotic and necrotic adhesion to sellar wall, incomplete tumor resection	Subtotal resection, radiotherapy (54 Gy)	Good
15	2024	Normal	Pituitary adenoma with apoplexy	Endoscopic transnasal skull base tumor resection	Resection	Good

**Table 3:** Differential diagnosis of pituitary tumors.

**Tumor Type**	**Population Distribution**	**Imaging Features**
Pituitary Adenoma	Adults; more common in middle-aged	Typically presents as a well-defined mass in the sellar region on MRI. It may appear hyperintense or hypointense on T1-weighted images and shows enhancement post-contrast. Often causes sellar enlargement.
Pituitary Carcinoma	Very rare; adults	Similar to adenomas but with evidence of metastasis. Usually more aggressive, larger at initial diagnosis, and may invade surrounding structures like the cavernous sinus.
Primary Pituitary Melanocytoma	Rare; can occur at any age, more common in adults.	Appears hyperintense on T1-weighted MRI and hypointense on T2-weighted MRI, mainly due to high melanin content. Shows marked enhancement post-contrast. Typically located in the sellar region, and may invade surrounding tissues.
Craniopharyngioma	Common in children and adults; two peaks in incidence (children and elderly)	Typically presents as a suprasellar cystic mass with calcifications on CT and MRI. The cyst content may appear hyperintense on T2-weighted images, with variable enhancement post-contrast.
Rathke's Cleft Cyst	All age groups; often an incidental finding	Appears as a non-enhancing cystic lesion in the sellar region, typically hypointense on T1-weighted images and hyperintense on T2-weighted images, with no solid components unless hemorrhage is present.
Metastatic Pituitary Tumor	Elderly patients with a history of cancer	Usually presents as a rapidly enlarging mass, often associated with diabetes insipidus. Commonly linked to known primary cancers like breast or lung cancer. Imaging may resemble an invasive adenoma.
Pituitary Hyperplasia	All age groups and is associated with conditions causing increased pituitary stimulation	Diffused enlargement of the pituitary without a distinct mass, typically showing uniform enhancement on MRI. Reflects physiological or pathological stimulation of the pituitary.

**Table 4:** Overview of immunohistochemical marker expression in primary pituitary melanocytoma.

**Case**	**Year\Refs.**	**Immunohistochemistry Results**										**Other Molecular Characteristics**	**Special Stains**
		**S-100**	**HMB-45**	**Melan-A**	**CD68**	**Vimentin**	**PS100**	**Anti-Keratin**	**Anti-EMA**	**Anti-CEA**	**MIB-1**		
1	1963 [7]	+	+	-	-	-	-	-	-	-	-	-	Melanoma tissue penetrates the lymphatic vessels of the pituitary capsule, and melanin granule staining is positive.
2	2015 [10]	+	+	+	+	+	-	-	-	-	3%	-	-
3	2017 [11]	+	-	-	+	-	-	-	-	-	<1%	-	-
4	2016 [16]	+	+	+	-	-	+	-	-	-	40%	BRAF V600 gene mutation.	HE staining positive.
5	1997 [17]	-	+	-	-	+	-	-	-	-	-	-	Masson-Fontana staining for melanin: +
6	2006 [18]	+	+	+	-	-	-	-	-	-	-	-	Masson-Fontana staining shows focal pigmentation.
7	1976 [19]	+	+	-	-	-	-	-	-	-	-	-	Masson- Masson-Fontana staining for melanin: + Melanin bleaching staining: +
8	2011 [20]	+	+	+	-	+	+	-	-	-	60%	-	-
9	2013 [21]	+	+	+	-	-	-	-	-	-	60%	Pituitary hormones or endocrine markers synaptophysin and chromogranin show no staining.	HE and PAS staining positive.
10	1990 [22]	-	-	-	-	-	-	-	-	-	-	-	Masson-Fontana staining for melanin: + Melanin bleaching staining: + Prussian blue staining: -
11	2004 [23]	+	+	+	-	-	-	-	-	-	15%-30%	-	Masson-Fontana staining for melanin: +
12	2023 [24]	+	-	-	-	-	-	+	-	-	<1%	-	Masson-Fontana staining for melanin: +
13	2023 [24]	+	+	-	-	-	-	-	-	-	1%	-	-
14	2009 [25]	+	+	-	-	-	-	-	-	-	-	-	-

**Table 5:** Detailed comparison of histological features of primary pituitary melanocytomas.

**Case**	**Year\Refs.**	**Description**	**Pigmentation**	**Cell Morphology**	**Nuclear Characteristics**	**Other Notable Features**
1	1963 [7]	Small round cells, sparse cytoplasm, with pigment granules	Present	Small round cells	Sparse cytoplasm	Pigment granules
2	2015 [10]	Bundles of spindle-shaped small-nucleus cells and eosinophilic cells	Sparse	Spindle-shaped, eosinophilic	Small nuclei	Presence of foam cells
3	2017 [11]	No apoptosis or mitoses	Absent	Epithelioid and spindle-shaped	Irregular	Densely nested arrangement
4	2016 [16]	Melanin-containing globoid cells, prominent nucleoli	Present	Globoid cells	Prominent nucleoli	-
5	1997 [17]	Dark pigmentation, high mitotic activity	Present	-	High mitotic activity	Yellowish-brown tumor
6	2006 [18]	All tumor markers are negative	-	-	-	Tumor markers negative
7	1976 [19]	Solid, adenomatous, papillary, rare mitoses	Present	Varied	Rare mitoses	Solid, adenomatous structures
8	2011 [20]	Eosinophilic cells, large and regular, no atypia	Present	Eosinophilic cells	Regular	-
9	2013 [21]	Highly vascular, lymphocyte infiltration, polymorphism	Absent	Polymorphic cells	Marked polymorphism	High vascularity, inflammation
10	1990 [22]	Marked polymorphism in permanent sections	Absent	Polymorphic cells	Marked polymorphism	-
11	2004 [23]	Partial necrosis, focal melanin, nuclear pleomorphism	Partially present	Polymorphic cells	Marked pleomorphism	Partial necrosis
12	2023 [24]	Cytoplasmic pigment-dense, focal papillary structures	Present	-	Round nuclei, prominent nucleoli	Papillary structures
13	2023 [24]	Positive for S-100 and Melan-A	-	-	-	Positive for S-100 and Melan-A
14	2009 [25]	Containing brown granules, eosinophilic cytoplasm	Present	Trabecular or nodule-like	-	Rich vascularity

## Data Availability

The data supporting the findings of the article are available within the article.

## LIST OF ABBREVIATIONS

CNS: Central Nervous System
MRI: Magnetic Resonance Imaging
CT: Computed Tomography
ADL: Activities of Daily Living
BPSRQ: Brief Psychosomatic Health Questionnaire
CTA: CT Angiography
CTV: CT Venography

## STANDARDS OF REPORTING

CARE guidelines were followed.
